# ‘Mental heAlth and well-being in rUgby pLayers’ (MAUL) study: an online survey of diverse cohorts of rugby union players internationally

**DOI:** 10.1136/bmjsem-2024-002164

**Published:** 2024-12-07

**Authors:** Steffan Griffin, Rebecca Syed Sheriff, Kathryn Dane, Kearnan Myall, Kaitlin Simpson, Heather Lewis, Caithriona Yeomans, Jon Patricios, Simon Kemp, Karim Khan, Debbie Palmer, Samantha Fawkner, Paul Kelly

**Affiliations:** 1 University of Edinburgh Physical Activity for Health Research Centre, Edinburgh, UK; 2 Medical Services, Rugby Football Union, Twickenham, UK; 3 Department of Psychiatry, Oxford University, Oxford, UK; 4 Oxford Health NHS Foundation Trust, Oxford, UK; 5 Department of Physiotherapy, Trinity College Dublin, Dublin, Ireland; 6 Mental Health Foundation, Cardiff, UK; 7 Irish Rugby Football Union, Dublin, Ireland; 8 Wits Institute for Sport and Health (WISH), University of the Witwatersrand Faculty of Health Sciences, Johannesburg, South Africa; 9 London School of Hygiene & Tropical Medicine, London, UK; 10 Centre for Aging SMART, The University of British Columbia Department of Family Practice, Vancouver, British Columbia, Canada; 11 Edinburgh Sports Medicine Research Network, Institute for Sport, PE and Health Sciences, University of Edinburgh, Edinbugh, UK; 12 Edinburgh Orthopaedics Sports Medicine Research, Edinburgh, UK

**Keywords:** Rugby, Well-being, Public health

## Abstract

**Introduction:**

Mental health and well-being is a relatively under-researched area in rugby, especially outside the elite men’s game. Evidence suggests that physical activity and sports benefit mental health and well-being, and rugby provides health-enhancing moderate-to-vigorous physical activity.

**Objective:**

This cross-sectional study used an online approach and engaged national rugby governing bodies to understand adult rugby players’ mental health and well-being and increase the diversity of the current evidence base.

**Results:**

500 rugby players completed an online survey. 44% of participants identified as female, and 55% as male. The UK (67%), Ireland (15%) and South Africa (12%) were the countries with the highest representation. 71% of participants were amateur players, with elite players making up 20% of the population. 87% of players participated in contact forms of the game, with 9% predominantly playing non-contact rugby. Over 50% of participants reported that rugby impacted ‘extremely’ positively on both their mental health and well-being. Based on the Kessler psychological distress scale (K10), 57.8% of all respondents belonged to the ‘psychologically well’ group. Males were more likely to belong to this group than females (p=0.01). Non-contact and amateur players had lower scores of psychological distress than contact and professional players (p=0.001 and p=0.006), respectively. Non-contact players had higher well-being (Short Warwick-Edinburgh Mental Well-being Scale) scores than contact players (p<0.001).

**Conclusion:**

This study provides new insights into the mental health and well-being of a diverse group of rugby players.

**WHAT IS ALREADY KNOWN ON THIS TOPIC**

- There is relatively limited research investigating mental health and well-being in rugby players. Of the published research, the majority has focused on elite male players who play contact forms of the sport.

**WHAT THIS STUDY ADDS**

- Across all playing groups, most respondents self-reported that rugby positively impacted their mental health and well-being.
- Male, amateur and non-contact players were found to have better mental health and well-being than female, professional and contact-playing players, respectively.

**HOW THIS STUDY MIGHT AFFECT RESEARCH, PRACTICE OR POLICY**

- This study has demonstrated a feasible approach to international research into mental health and well-being in diverse rugby playing groups, including women’s, non-contact and amateur players.

## Introduction

 Rugby union, including adapted forms of the game such as wheelchair rugby (henceforth referred to as ‘rugby’) is played in over 130 countries by more than 8 million people.^1^ Most scientific research in rugby centres around injury and illness epidemiology and prevention,^2^,^6^ and most research into rugby players’ health and well-being focuses on physical fitness or physical health.^7^

Evidence suggests an increasing prevalence of mental health problems in the world’s population, with the WHO recently issuing a ‘wake-up call to all countries to pay more attention to mental health’ following a sharp increase in the number of people living with anxiety and depressive disorders in the first year of the COVID-19 pandemic.^8^ Physical activity and sport are known to be beneficial for mental health and well-being,^9 10^ and while rugby is known to provide health-enhancing moderate-to-vigorous physical activity,^11^ the mental health and well-being status of participants as well as the perceived impact of participation are less understood.^7^

Existing studies have predominantly focused on the symptomology of elite male players who played contact forms of the game.^712^,^14^ Relatively fewer studies have outlined the mental health and well-being status of female, amateur, non-contact and wheelchair rugby players or explored players’ self-reported perception of the impact of rugby on their mental health and well-being. Women’s rugby is especially relevant to policymakers, given that it is the major driver of new growth for the game.^1^

We designed three research questions below to improve the understanding of rugby players’ mental health and well-being and to increase the diversity of the current evidence base.

1. What self-reported effect does rugby have on players’ mental health and well-being, and does this vary by gender, type of rugby, and level of participation?
2. What is the mental health and well-being status of those who play rugby, and does this vary by gender, type of rugby, and level of participation?
3. What are the perceived mechanisms underlying the effect rugby has on players’ mental health and wellbeing?

## Methods

### Study design

This was a cross-sectional study of adult (aged 18 and over) rugby players. The Strengthening the Reporting of Observational Studies in Epidemiology Statement for reporting cross-sectional studies was followed.

### Ethics approval

Ethics approval was provided by the University of Edinburgh’s Moray House School of Education and Sport Ethics Subcommittee (REF SGRI21012022). Sponsorship was provided by the University of Edinburgh (REF CAHSS2202/07). The study also gained local ethical approval from the Irish Rugby Football Union’s research committee (REF 05–22) as part of the terms of engagement. Local ethical approval was not sought as part of the engagement process with the South Africa Rugby Union as the survey was online-based, and as such exempt.^15^

### Patient and public involvement (PPI)

We sought feedback on the questionnaire design and methods from 15 international male and female experts involved in playing or coaching rugby or rugby-related research. The lead author identified these individuals. The group provided feedback on the survey design, incorporating comments into the final questionnaire.

### Setting, sampling and participants

The national governing bodies of England, Ireland and South Africa approved the dissemination of the questionnaire to several of their representative teams and clubs across various levels of play.

To attain as diverse a set of participants as possible, we designed a sampling matrix of rugby settings (clubs, teams or organisations) to recruit from (online supplemental appendix 1). This was used by governing bodies to purposively identify relevant settings. Introductions were made to a local ‘coordinator’ (usually a coach, healthcare professional or committee member), who was then assigned to share the questionnaire. They were sent a template to disseminate to their adult players with reminders at 2-and-4 weeks.

Additionally, the networks of the author team were used to contact relevant stakeholders with links to more diverse rugby settings, who were recruited as local coordinators with the same approach outlined above. Following data collection across the various settings, each local coordinator was asked to provide a sample size to allow us to calculate a response rate.

The inclusion criteria for participants were: (i) aged 18 years and over; (ii) actively registered as players or considered themselves as active players in a form of rugby union including wheelchair rugby and (iii) able to read and understand the English language.

### Data collection

We collected data from July to October 2022 after a successful pilot study in a rugby club familiar to the lead researcher (done to ensure that the online format was user-friendly). Participants completed an online non-identifiable anonymised questionnaire on the Qualtrics (Qualtrics, Provo, Utah) platform, which included a patient information sheet and consent form (online supplemental appendix 2). The questionnaire took approximately 15 min and comprised demographic questions followed by self-reported mental health and well-being measures.

Participants then completed the Kessler Psychological Distress Scale (K10) and Short Warwick-Edinburgh Mental Well-being Scale (SWEMWBS). The University of Warwick (ID: 547861904) provided access to the SWEMWBS.^16^ These questionnaires are validated measures of population health and well-being^17^ previously used in sports settings.^18^,^20^ Literature correlates K10 and SWEMWBS outcomes with clinical ratings of mental health and well-being.^21^,^24^

K10 scores are based on a scale from 10 to 50 and have been categorised into various levels of psychological distress (table 1).^25^

**Table 1:** K10 score groupings and categorisation^25^

K10 total score levels	Level of psychological distress
10–19	The score indicates that the client or patient may not be experiencing significant distress.
20–24	The client or patient may be experiencing mild levels of distress consistent with a diagnosis of mild depression and/or anxiety disorder.
25–29	The client or patient may be experiencing moderate levels of distress consistent with a diagnosis of moderate depression and/or anxiety disorder.
30–50	The client or patient may be experiencing severe levels of distress consistent with a diagnosis of severe depression and/or anxiety disorder.

Higher SWEMWBS scores indicate higher mental well-being; total scores are transformed into metric scores.^23^ Though there are no clinically validated diagnostic cut-offs, the authors of the SWEMWBS suggest that scores of >20 correspond to psychologically ‘well’ groups.^17 26^

Questions and predetermined options relating to mechanisms and facilitators of mental health and well-being in rugby were designed in consultation with the patient and public involvement (PPI) group (online supplemental appendix 2).

Electronic data were securely stored in a restricted access folder on the University of Edinburgh Datastore site following current General Data Protection Regulations (GDPR).

### Statistical analysis

Statistical analysis and presentation adhered to the recommendations outlined in the CHecklist for statistical Assessment of Medical Papers.^27^ We performed statistical analyses on the data of all participants who provided a completed survey. We kept all cases in the overall analysis regardless of the sample size in certain subcategories. Still, it was decided that not all subcategory data would be presented in tables if the sample size was small (eg, outcomes relating to ‘other’ gender, n=3).

We used Q-Q plots and Shapiro-Wilk tests to calculate normality. Although the outcomes did not perfectly follow a normal distribution, we used parametric tests to test for group differences, given the large sample size and the fact that the data broadly followed a normal distribution.

We summarised demographic variables (mean (SD) or n (%)) by group. Means and 95% CIs for each cohort were estimated by separate independent t-tests or analysis of variance (ANOVA) models for K10 and SWEMWBS scores. If results showed an expected cell count <5 in over 20% of cells during analysis, some outcomes were combined to form a binary outcome (eg, responses grouped into either a ‘positive’ self-reported impact (a combination of extremely and somewhat positive) or ‘not positive’ self-reported impacts (all other answers)). We compared groups using independent t-tests or ANOVA for continuous variables or χ2 tests for categorical variables. Where relevant, we also used binomial regression to determine ORs between independent groups for binary dependent outcomes (which were formulated by collapsing outcome categories).

We conducted all analyses within SPSS V.29 (Statistical Package for Social Sciences), with significance at p<0.05. Given that research in this cohort was exploratory, we deemed it appropriate not to conduct a sample size calculation.^28^

## Results

### Response rate

We recruited 42 ‘coordinators’ to distribute the survey, and 28 provided us with the total sample size. No final confirmation of sample size was obtained from 14 coordinators. We know from the 28 coordinators who responded (11 were based in England, 9 in Ireland, 6 in South Africa and two in ‘other’ countries), that 1562 active adult rugby players were sent the survey, and 456 responses were received, providing a response rate of 29.2%. Response rates were broadly consistent across all these settings, with no obvious outliers.

### Descriptive characteristics

A total of 565 adult players started the survey, with 500 players voluntarily completing it (88% completion). Demographic data are provided in table 2.

**Table 2:** Demographics for MAUL population (n=500)

Demographic variables	Mean	SD
Age	29.6	11.2
Years of participation	13	10.5
Gender		
	**N**	**%**
Male	275	55%
Female	222	44.4%
Other	3	0.6%
Ethnicity		
	**N**	**%**
Asian: Chinese	2	0.4%
Black African or Caribbean	22	4.4%
Other Asian background	2	0.4%
Other Black/African/Caribbean	6	1.2%
Other ethnic group	10	2%
Other mixed/multiple ethnic backgrounds	7	1.4%
Other white background	25	5%
White and Asian	5	1%
White and Black African or Caribbean	9	1.8%
White: Australasian	4	0.8%
White: European	408	81.6%
Current geographic location—by country		
	**N**	**%**
France	1	0.2%
Hong-Kong	2	0.4%
Ireland	75	15%
Laos	1	0.2%
South Africa	60	12%
UK	337	67.4%
Other	24	4.8%
Participation status		
	**N**	**%**
Elite	102	20.4%
Semiprofessional	45	9%
Regular amateur*	315	63%
Recreational amateur*	38	7.6%
What form of rugby played most?		
	**N**	**%**
Contact	434	86.6%
Non-contact	46	9.2%
Wheelchair	10	2%
Other	10	2%

* Definition for regular participation was playing or training twice or more a month on average.

MAULMental heAlth and well-being in rUgby pLayers

### Self-reported impact of rugby on participants’ mental health

Nearly 90% of participants reported that rugby had an ‘extremely’ or ‘somewhat’ positive impact on their mental health (table 3).

**Table 3:** Self-reported impact of rugby on mental health by different demographic independent variables

	Self-reported impact of rugby on mental health				
	Extremely negative impact	Somewhat negative impact	No impact or equally positive and negative impact	Somewhat positive impact	Extremely positive impact
Total (n=500)	0.2%	4.0%	7.8%	39.4%	48.6%
Male (n=275)	0.4%	3.6%	7.6%	36.0%	52.4%
Female (n=222)	0.0%	4.5%	8.1%	43.7%	43.7%
Contact (n=434)	0.2%	4.6%	8.8%	40.3%	46.1%
Non-contact (n=46)	0.0%	0.0%	2.2%	37.0%	60.9%
Elite (n=102)	1.0%	9.8%	13.7%	49.0%	26.5%
Semiprofessional (n=45)	0.0%	2.2%	11.1%	33.3%	53.3%
Regular amateur (n=315)	0.0%	2.9%	6.0%	37.8%	53.3%
Recreational amateur (n=38)	0.0%	0.0%	2.6%	34.2%	63.2%

The odds ratioOR for amateur players (both regular and recreational players) reporting some form of positive impact on their mental health compared tocompared with professional players (both elite and semiprofessional) was 2.986 (95% CI 1.724 to 5.170).

There was no statistically significant difference in the self-reported impact of rugby between males and females, χ^2^(4, n=497)=4.820, p=0.306). Non-contact players were more likely to report a positive impact of rugby on their mental health than contact players (χ^2^(1, n=480)=4.96, p=0.026).

### Self-reported impact of rugby on participants’ mental well-being

Over half of the participants reported that rugby had an ‘extremely positive’ impact on their mental well-being (table 4). There was no statistically significant difference between male and female participants, χ^2^(3, n=497)=0.836, p=0.841).

**Table 4:** Self-reported impact of rugby on mental well-being by different demographic independent variables

	Self-reported impact of rugby on mental well-being				
	Extremely negative impact	Somewhat negative impact	No impact or equally positive and negative impact	Somewhat positive impact	Extremely positive impact
Total (n=500)	0%	4.8%	6%	38.6%	53.6%
Male (n=275)	0%	1.5%	5.5%	39.3%	53.8%
Female (n=222)	0%	2.3%	6.8%	38.3%	52.7%
Contact (n=434)	0%	1.8%	6.9%	39.9%	51.4%
Non-contact (n=46)	0%	0%	0%	30.4%	69.6%
Elite (n=102)	0%	3.9%	11.8%	45.1%	39.2%
Semiprofessional (n=45)	0%	0%	0%	40%	60%
Regular amateur (n=315)	0%	1.6%	5.7%	36.2%	56.5%
Recreational amateur (n=38)	0%	0%	0%	39.5%	60.5%

### Kessler Psychological Distress Scale (K10) results

Over half (57.8%) of all respondents belonged to the ‘psychologically well’ group (table 5). Males and non-contact players were more likely to have a score consistent with being psychologically well compared with females (χ^2^(3, n=500)=11.155, p=0.011) and contact players, respectively (χ^2^(3, n=480)=12.368, p=0.006).

**Table 5:** Proportional representation of MAUL participants in respective K10 clinical categories

	K10 category (score banding)			
	Psychologically well or likely to be well (0–19)	Mild psychological distress or likely to have a mild m–24)	Moderate psychological distress or likely to have a moderate mental disorder (25–29)	Severe psychological distress or likely to have a severe mental disorder (30–50)
Total (n=500)	57.8%	20.6%	8.8%	12.8%
Male (n=275)	62.9%	20.7%	5.8%	10.5%
Female (n=222)	51.4%	20.7%	12.2%	15.8%
Contact (n=434)	54.4%	21.9%	9.4%	14.3%
Non-contact (n=46)	80.4%	13.0%	4.3%	2.2%
Elite (n=102)	48.0%	26.5%	8.8%	16.7%
Semiprofessional (n=45)	48.9%	33.3%	4.4%	13.3%
Regular amateur (n=315)	58.1%	18.4%	10.5%	13.0%
Recreational amateur (n=38)	92.1%	7.9%	0.0%	0.0%

The odds ratioOR for amateur players (regular and recreational amateur combined) belonging to the ‘psychologically well’ category compared tocompared with professional players (elite and semiprofessional combined) was 1.729 (95% CI 1.172 to 2.549).

MAULMental heAlth and well-being in rUgby pLayers

### Short Warwick-Edinburgh Mental Well-being Scale (SWEMWBS)

The mean SWEMWBS score was 22.36±3.69 (table 6). Non-contact participants had a statistically significant higher mean SWEMWBS score (non-contact=24.93, SD=5.26) compared with their contact counterparts ((contact=21.88, SD=3.21), t(478)=−5.703, p<0.001) implying non-contact players had higher levels of well-being.

**Table 6:** Mean SWEMWBS scores by demographic variables

	Mean SWEMWBS scores±SD
Total (n=500)	22.36±3.69
Male (n=275)	22.26±3.66
Female (n=222)	22.49±3.75
Contact (n=434)	21.88±3.21
Non-contact (n=46)	24.93±5.26
Elite (n=102)	22.73±4.09
Semiprofessional (n=45)	23.45±4.22
Regular amateur (n=315)	22.04±3.54
Recreational amateur (n=38)	22.77±2.81

SWEMWBSShort Warwick-Edinburgh Mental Well-being Scale

### Insights into potential mechanisms and facilitators

Participants’ most frequently chosen mechanisms underlying any positive effect that rugby might have had on their mental health or well-being options were providing fun (87.2%), providing a form of physical activity (85.2%), increasing fitness (77.8%), providing a social environment and support (71.6%) and being outdoors (71.4%).

Participants’ most frequently chosen mechanisms underlying any negative effect that rugby might have had on their mental health or well-being options were injuries including sprains, strains and fractures (14.2%), a pressure to perform (10.6%), not being selected (8.6%), head injuries/concussions (7.4%) and exposure to aggressive coaches (5.2%).

When participants were asked to rank the biggest influencers on their mental health (either positively or negatively) within the rugby environment, teammates (97.2%) and coaches (94.4%) ranked highest, followed by parents/guardians (n=208, 41.6%).

## Discussion

### Principle findings

This study aimed to understand the mental health and well-being of rugby players across a wider demographic and to increase the diversity of the current evidence base. We found across an international, diverse cohort of rugby players, most self-reported that rugby positively influenced their mental health and well-being. Nearly 60% of participants also had a K10 score corresponding with being ‘psychologically well’.

### Self-reported effect of rugby on players’ mental health and well-being

Most participants reported that rugby positively impacted their mental health and well-being. Non-contact rugby players were likelier to report that rugby positively impacted their mental health and well-being than contact rugby players. To our knowledge, this difference between contact and non-contact rugby players has not been reported previously in the literature. It is also reinforced by a significant difference in the mean SWEMWBS scores of both groups, though these differences should be caveated by the almost 10:1 ratio of players in the respective categories, which limits our ability to draw firm, generalisable conclusions.

Further research could look to explore this in greater detail, including potential mechanism(s) to explain these differences. This could be due to non-contact rugby players benefiting from all the positive benefits associated with team sport but without exposure to potential negative aspects (such as risk of injury). A 2021 study found that retired elite rugby players scored consistently worse for psychological signs of depression, anxiety and irritability when compared with amateur rugby code and non-contact athlete groups, but there were no statistically significant differences between amateur rugby and non-contact athletes.^29^ There is a paucity of other studies investigating this difference in the literature, with most research comparing team sports as a whole to individual sports, with the former having ‘more potent and additional benefits for mental and social outcomes’ compared with the latter.^9^

### Validated mental health and well-being outcomes

Based on the K10 scores, nearly 60% of participants could be considered ‘psychologically well’, with males and non-contact players more likely to belong in this category than females and contact players. Similarly, we found that amateur players (regular and recreational amateur combined) were almost twice as likely to belong to the ‘psychologically well’ category compared with professional players (elite and semiprofessional combined). These results are in keeping with the literature showing that females have generally higher rates of anxiety than males.^30^,^32^ In our cohort of female rugby players, there was a lower proportion of amateur players (65%) than male players (75%), which could also contribute to this gender difference.

Our results are in keeping with studies that show that the prevalence of mental health symptoms and disorders in elite athlete populations is slightly higher than in the general population.^33 34^ This is likely related to the sport-specific stressors that elite athletes are exposed to over the course of their careers, and interestingly these differences can persist into retirement.^33^

Our cohort had a lower prevalence of clinically significant levels of mental distress (defined as K10>19) compared with a 2020 study of nearly 12 000 adults in the UK, with 42.2% of our cohort meeting this definition compared with 56.4% in the general population.^35^

When we analysed the SWEMWBS scores, the only statistically significant intergroup difference was in the form of rugby most played, where non-contact players had higher levels of well-being compared with contact players.

When comparing mean scores to other population groups, the results are similar to a UK study of over 27 000 adults that reported a mean score of 23.7 for men and 23.2 for women.^31^ One study of 233 elite rugby league players in England reported a mean SWEMWBS score of 25.07, indicating potentially slightly higher levels of well-being in their cohort than ours.^20^ However, the clinical significance of this difference is unclear.^17 26^

### Plausible explanations/mechanisms

It is well documented that physical activity provides mental health and well-being benefits,^8 9^ which our participants identify as one of the main mechanisms underlying a largely positive self-reported effect of participation on mental health and well-being. Social interaction has also been cited as a facilitator of good mental health among amateur football players^36^ and was also found to be a commonly cited facilitator here.

The finding that amateur players were nearly three times more likely to report that rugby positively influenced their mental health than professional players could be attributed to the fact that ‘fun’ was the most selected rugby-related facilitator of positive mental health and well-being among participants. It would seem feasible that fun might be less experienced in professional environments.

Conversely, the most selected negative facilitators of mental health and well-being were injuries, pressure to perform and not being selected. Professional players are known to have a higher injury burden than their amateur counterparts,^2 7^ which would support this difference, and it could be argued that pressure and selection-related anxieties might be more prevalent and intense in more professional environments (due to contract negotiations, pressure to succeed and career prospects, etc).

### Strengths

Our study provides insights into the mental health and well-being of populations where research gaps currently exist, for example, in women’s and amateur rugby. It also uses self-reported and validated clinical instruments to provide insights into participants’ mental health and well-being.

Although we reached more diverse groups than in previous studies, there were relatively low numbers of non-contact and wheelchair rugby players in this study. This somewhat limits the applicability of the results to these rugby-playing cohorts.

Due to the nature of the study and involving local coordinators with no direct obligation to provide sample sizes, response rates could only be calculated in two-thirds of the study’s settings. The overall response rate of 29.2% is comparable to that obtained in similar studies,^13^ though it falls below the average online survey response rate of 44.1%.^37^ However, given that this cross-cultural international study involves cohorts where stigma may exist around mental health^38^ and where men especially have been considered to resist talking and under-report their mental health,^39^ this could alternatively be seen as a strength of the study’s design.

### Limitations

Some biases need to be considered in the context of the results. The main limitation is that, despite efforts to maximise the diversity of our participants, the majority of our results come from countries where rugby is well-established as one of the most popular sports. As such, applicability to other settings where this is not the case is limited and should be considered by readers.

To minimise the risk of a non-response bias, we piloted a questionnaire in a rugby club in England, and the high response rate (76%) in this setting provided reassurance that we had tried to minimise this type of bias as much as possible.

Through consultation with our PPI group, we also spent a significant amount of time reviewing the wording of the questionnaire to minimise the risk of any confirmation, extreme or neutral response bias. We also emphasised the anonymous nature of the questionnaire to try and further minimise the risk of confirmation and social desirability bias. In questions where respondents could choose from multiple options, we ensured the order was randomised to try and minimise the risk of primacy bias.

The present study was cross-sectional in nature and asked the participants to rate aspects of their mental health based on recent periods of time. As such, given the range of factors that influence mental health, it should be considered that these influences and participants’ contexts may have changed since the time of the questionnaire, which could affect the applicability of the results.

### Recommendations for research

This study provides previously unpublished insights that show that rugby is considered by participants to affect their mental health and well-being positively. We have also found some differences in the mental health and well-being of various subgroups of rugby players. Future research could look to perform targeted research in these cohorts to try and determine some of the underlying mechanisms and reasons for the differences reported and use different research designs (including qualitative approaches) to capture more rich data around participants’ lived experiences and facilitators of mental health and well-being.

### Implications for practice and policy

We found higher levels of well-being in amateur and non-contact rugby players compared with professional and contact rugby, which policymakers might feel highlights the types of rugby with the greatest public health relevance. Overall, the levels of psychological distress and well-being are comparable and, in some cases, more favourable to those of the general population. However, these studies have different population characteristics that render a direct comparison impractical.

This study can provide policymakers with focus areas to further positively influence participants’ mental health and well-being by embracing the positive facilitators and avoiding/minimising exposure to negative facilitators. With coaches and teammates identified as the biggest influences on mental health and well-being, policymakers could engage with these groups to influence participants’ mental health and well-being.

## Conclusion

This study provides new insights into the mental health and well-being of a diverse group of rugby players. Most participants self-reported that rugby positively affected their mental health and well-being. Male, amateur and non-contact players had lower scores of psychological distress compared with their female, professional and contact-playing counterparts, respectively. Non-contact rugby players were also found to have higher levels of well-being than contact rugby players. However, the relatively lower number of non-contact players limits the generalisability of this finding.

Future research is needed to investigate further the underlying positive and negative facilitators of mental health and well-being in rugby players. Still, this study provides insights into research questions relating to mental health and well-being among traditionally under-represented groups of rugby players worldwide.

## supplementary material

10.1136/bmjsem-2024-002164 online supplemental file 1

10.1136/bmjsem-2024-002164 online supplemental file 2

## Data Availability

All data relevant to the study are included in the article or uploaded as supplementary information.
