# Supplementary material for: ‘Mental heAlth and well-being in rUgby pLayers’ (MAUL) study: an online survey of diverse cohorts of rugby union players internationally
Source: BMJ Open Sport Exerc Med. 2024 Dec 7;10(4):e002164. doi: 10.1136/bmjsem-2024-002164 (PMC11629002; doi:10.1136/bmjsem-2024-002164)
Supplement: online supplemental file 1 [file bmjsem-10-4-s001.pdf]

Appendix 1. MAUL Sampling Matrix.

		Contact		Non-Contact		Wheelchair	
		Male	Female	Male	Female	Male	Female
Setting	High-Income Country (HIC)						
	Low-Middle Income Country (LMIC)						
Level	Elite						
	Recreational						
Geography	Urban						
	Rural						
