# Supplementary material for: ‘Mental heAlth and well-being in rUgby pLayers’ (MAUL) study: an online survey of diverse cohorts of rugby union players internationally
Source: BMJ Open Sport Exerc Med. 2024 Dec 7;10(4):e002164. doi: 10.1136/bmjsem-2024-002164 (PMC11629002; doi:10.1136/bmjsem-2024-002164)
Supplement: online supplemental file 2 [file bmjsem-10-4-s002.pdf]

PARTICIPANT CONSENT FORM

Study Title: **Mental heAlth and wellbeing in rUgby pLayers (MAUL)**

Researcher's name and contact details: Dr Steffan Griffin
s1135547@ed.ac.uk

Please tick box

1. I confirm that I have read and understood the Participant Information Sheet (Version 1.0 dated 22/02/2022) for the above study. ☐

2. I have been given the opportunity to consider the information provided, ask questions and have had these questions answered to my satisfaction. ☐

3. I understand that my participation is voluntary and that I can withdraw from the study at any time. ☐

4. I understand that my anonymised data will be stored long-term (for a minimum of 5 years) on a secure University of Edinburgh database and may be used in future ethically approved research. ☐

5. I understand that the results of the study will be shared with individuals from the University of Oxford, the Rugby Football Union, and World Rugby. ☐

6. I understand that the relevant sections of my data collected may be looked at by individuals from the Sponsor (University of Edinburgh), where it is relevant to my taking part in the research. I give permission for these individuals to have access to my data.

7. By ticking this box I agree to take part in the above study. ☐

PARTICIPANT INFORMATION SHEET

Mental heAlth and wellbeing in rUgby pLayers (MAUL)

You are being invited to take part in research on mental health and wellbeing in rugby players. Dr Steffan Griffin (PhD student) at the University of Edinburgh is leading this research. Before you decide whether to take part it is important you understand why the research is being conducted and what it will involve. Please take time to read the following information carefully.

WHAT IS THE PURPOSE OF THE STUDY?

The purpose of the study is to discover how rugby participation influences mental health and wellbeing, and to find out whether rugby could be doing more to positively influence the mental health and wellbeing of those who play the game. This will be one of the first studies in the world to look into this, and findings will be shared with governing bodies and policymakers so that they can be acted upon and disseminated.

WHY HAVE I BEEN INVITED TO TAKE PART?

You are invited to participate in this study because you have indicated that you are an English-speaking adult (aged 16+) that plays or has played a form of rugby union.

DO I HAVE TO TAKE PART?

No – it is entirely up to you. If you do decide to take part, you are still free to withdraw at any time and without giving a reason. Deciding not to take part or withdrawing from the study will not affect your involvement in rugby.

If you decide to take part, you may leave the survey at any time. As participation is anonymous, it is not possible to withdraw your data once you submit your survey responses.

WHAT WILL HAPPEN IF I DECIDE TO TAKE PART?

If you do decide to take part, please keep this Information Sheet. You will be asked to sign an Informed Consent Form online to show that you understand your rights in relation to the research, and that you are happy to participate.

You will be asked a number of questions regarding your participation in rugby, your perceptions around how rugby influences your mental health and wellbeing, and finally your self-perceived mental health and wellbeing. The online questionnaire should take around 10-minutes to complete.

WHAT ARE THE POSSIBLE BENEFITS OF TAKING PART?

There are no direct benefits, but by sharing your experiences with us, you will be helping the research team as well as governing bodies and policymakers to better understand any association between rugby and mental health and wellbeing, as well as how the sport can be optimised to positively influence this.

ARE THERE ANY RISKS OR DISADVANTAGES ASSOCIATED WITH TAKING PART?

There are no significant risks associated with participation. If you have symptoms of mental health challenges or should this survey exacerbate any mental health related symptoms, we encourage you to seek support through your usual channel(s), or through the appropriate resources in your region, such as below:

United Kingdom -NHS Mental Health Resources:
http://www.nhs.uk/Conditions/stress-anxiety-depression/Pages/mental-health-helplines.asp

WILL MY TAKING PART BE KEPT CONFIDENTIAL?

All the information we collect during the course of the research will be kept confidential and there are strict laws which safeguard your privacy at every stage.

HOW WILL WE USE INFORMATION ABOUT YOU?

We will need to use information from you for this research project.

This information will include your:

- Gender
- Age
- Ethnicity
- Employment Status

We will also be collecting data around your participation in rugby, your perceptions on how rugby influences health and wellbeing, and self-reported data on your own mental health and wellbeing. Your data will be referred to by a unique participant number rather than by name (which we will not be collecting).

We will keep all information about you safe and secure.

Your data will primarily be viewed by the research team at the University of Edinburgh, but the anonymised results will be shared with collaborators at the University of Oxford, the Rugby Football Union and World Rugby.

All electronic data will be stored in a restricted access folder on the University of Edinburgh Datastore site. Access will be restricted according to Datastore permissions, administered by the University of Edinburgh Digital Services team.

The University of Edinburgh is the sponsor for this study based in the United Kingdom. We will be using information from you in order to undertake this study and will act as the data controller for this study. This means that we are responsible for looking after your information and using it properly. The University of Edinburgh will keep identifiable information about your anonymised data for a minimum of 5 years.

.

WHAT ARE YOUR CHOICES ABOUT HOW YOUR INFORMATION IS USED?

- You can stop being part of the study at any time, without giving a reason, but we will keep information about you that we already have.
- We need to manage your records in specific ways for the research to be reliable. This means that we won't be able to let you see or change the data we hold about you.

WHERE CAN YOU FIND OUT MORE ABOUT HOW YOUR INFORMATION IS USED?

You can find out more about how we use your information at https://www.ed.ac.uk/records-management/privacy-notice-research

- by asking one of the research team
- by sending an email to s1135547@ed.ac.uk

WHAT WILL HAPPEN WITH THE RESULTS OF THIS STUDY?

The results of this study may be summarised in published articles, reports and presentations. You will not be identifiable from any published results. With your consent, your anonymised information may also be kept for future research and used in combination with research performed by the governing bodies to inform policy around participation. A summary of the findings from the study will be made available to participants who indicate they would like to receive this. Participants will be provided with Dr Griffin's email address which they can contact should they like to be updated with the results of the study.

WHO IS ORGANISING AND FUNDING THE RESEARCH?

This study has been organised by Dr Steffan Griffin. The study is being funded by World Rugby.

WHO HAS REVIEWED THE STUDY?

The study proposal has been reviewed and received a favourable opinion by the Moray House School of Education and Sport's Ethics Committee.

WHO CAN I CONTACT?

If you have any further questions about the study, please contact the lead researcher, Dr Steffan Griffin (s1135547@ed.ac.uk).

If you would like to discuss this study with someone independent of the study, please contact MHSES-Ethics@ed.ac.uk or the University's Data Protection Officer (for data-protection queries that cannot be addressed by researchers conducting study) dpo@ed.ac.uk

If you wish to make a complaint about the study, please contact: Research Governance Team (cahss.res.ethics@ed.ac.uk)]

Mental heAlth and wellbeing in rUgby pLayers (MAUL) survey

Start of Block: Demographic

How old are you currently (in years)? (dropdown)

What is your gender?

◯ Male

◯ Female

◯ Non-binary/third gender

◯ Prefer not to say

What is your ethnicity?

◯ White: European

◯ White: Australasian

◯ Other White background

◯ Black African or Caribbean

◯ Other Black/African/Caribbean background

◯ Pacifica

- ◯ Māori
- ◯ Indigenous Australian
- ◯ White and Black African or Caribbean
- ◯ White and Asian
- ◯ Other Mixed/Multiple ethnic background
- ◯ Asian: Indian/Pakistani/Bangladeshi
- ◯ Asian: Chinese
- ◯ Other Asian background
  - ◯ Other ethnic group ____
- ◯ I'd rather not say

In which country do you currently reside? (dropdown)

What is your current employment status?

- ◯ Full-time paid employment
- ◯ Part-time paid employment

◯ Full-time home maker

◯ Employed, but currently on sick leave

◯ Employed, but currently on maternity/paternity leave

◯ Self-employed

◯ Unemployed and not working

◯ Fully retired

◯ Retired, but still in paid employment

◯ Student

◯ Other ____

◯ I'd rather not say

End of Block: Demographics

Start of Block: Rugby Exposure

Do you currently play a form of rugby union?

◯ Yes

◯ No

Display This Question:

If Do you currently play a form of rugby union? = Yes

How would you generally describe your participation status in rugby over the period of time you have been involved?

○ Elite (Full-time job, or you receive payment/expenses and play at international level or the highest domestic level in your country)

○ Semi-professional (receive payment/expenses - play or train twice or more a month on average but not at the highest domestic level in your country)

○ Regular amateur (unpaid - play or train twice or more a month on average)

○ Recreational amateur (unpaid - play or train less than twice a month on average)

Display This Question:

If Do you currently play a form of rugby union? = Yes

What form of rugby do you play most?

○ Contact (XVs, 10s, 7s etc)

○ Non-contact (Tag, touch etc)

○ Wheelchair Rugby

○ Other ____

Display This Question:

If Do you currently play a form of rugby union? = Yes

For how many years have you played rugby union? (dropdown 1-99)

Display This Question:

If Do you currently play a form of rugby union? = Yes

How many times per week are you typically involved in rugby training? (i.e 'on-field' sessions and not including strength & conditioning training outside of the rugby setting) - DROPDOWN

Display This Question:

If Do you currently play a form of rugby union? = Yes

How many times per week do you play in rugby matches? - DROPDOWN

Display This Question:

If Do you currently play a form of rugby union? = No

Have you previously played a form of rugby union?

◯ Yes

◯ No

Skip To: End of Survey If Have you previously played a form of rugby union? = No

Display This Question:

If Have you previously played a form of rugby union? = Yes

How would you generally describe your participation status in rugby over the period of time you were involved?

◯ Elite (Full-time job, or you receive payment/expenses and play at international level or the highest domestic level in your country)

◯ Semi-professional (receive payment/expenses - play or train twice or more a month on average but not at the highest domestic level in your country)

◯ Regular amateur (unpaid - play or train twice or more a month on average)

◯ Recreational amateur (unpaid - play or train less than twice a month on average)

Display This Question:

If Have you previously played a form of rugby union? = Yes

What form of rugby did you play most often?

◯ Contact (XVs, 10s, 7s etc)

◯ Non-contact (Tag, touch etc)

◯ Wheelchair Rugby

◯ Other ____

Display This Question:

If Have you previously played a form of rugby union? = Yes

For how many years did you play rugby union? - DROPDOWN

Display This Question:

If Have you previously played a form of rugby union? = Yes

How many times per week were you typically involved in rugby training? (i.e 'on-field' sessions and not including strength & conditioning training outside of the rugby setting) - DROPDOWN

If Have you previously played a form of rugby union? = Yes

How many times per week did you play in rugby matches? - DROPDOWN

End of Block: Rugby Exposure

Start of Block: Health and Wellbeing Perceptions

Please select the most appropriate response to the questions below:

	Extremely negative impact overall	Somewhat negative impact overall	No impact or equally positive and negative impact	Somewhat positive impact overall	Extremely positive impact overall

What impact do you feel rugby has had overall on your physical fitness	○	○	○	○	○
What impact do you feel rugby has had overall on your physical health	○	○	○	○	○
What impact do you feel rugby has had overall on your mental health	○	○	○	○	○
What impact do you feel rugby has had overall on your wellbeing	○	○	○	○	○

Display this question

If Please select the most appropriate response to the questions below: - No impact or equally positive and negative impact Is Selected

Or Please select the most appropriate response to the questions below: - Somewhat positive impact overall Is Selected

Or Please select the most appropriate response to the questions below: - Extremely positive impact overall Is Selected

If you feel rugby has provided you with some form of benefit, by which mechanism(s) do you think playing rugby has done so? Please select all that apply

☐ Providing a form of physical activity

☐ Improving body composition

☐ A release from ordinary life

☐ Increasing fitness

☐ Providing a social environment and support

☐ Providing a purpose

☐ An opportunity for self-improvement

☐ Providing networking opportunities

☐ Being outdoors

☐ Providing a non-judgmental environment

☐ Respect from peers

☐ Social status

☐ An opportunity to exhibit skills and learning

☐ Fun

☐ I enjoy the competition

☐ Other (please specify) ________

In what ways could rugby contribute further to your (or others') health and wellbeing? Please select all that apply

☐ More of a focus on physical activity and/or fitness

☐ Provide education on health and wellbeing

☐ Further develop the social environment

☐ Proving formal networking opportunities

☐ Trying to separate community and elite rugby

☐ Further efforts to support equity and inclusion

☐ Reduce injury risk

☐ Less contact in training/matches

☐ Less focus on competition

☐ Less of an alcohol culture

☐ Other (please list)

____

Display this question

If Please select the most appropriate response to the questions below: - Extremely negative impact overall Is Selected

Or Please select the most appropriate response to the questions below: - Somewhat negative impact overall Is Selected

Or Please select the most appropriate response to the questions below: - No impact or equally positive and negative impact Is Selected

If you feel rugby has provided you with some form of negative impact, how do you think playing rugby has done so? Please select all that apply

☐ Head Injuries/Concussions

☐ Other Injuries (sprains/strains/fractures etc)

☐ Not being selected

☐ Being shouted at

☐ Culture of excess alcohol intake ± smoking

☐ Pressure to perform

☐ Exposure to aggressive coaches

☐ Excessive banter or bullying

☐ Other (please list)

____

In the setting that you play or have played rugby, who are/were the biggest ‘influencers’ of your mental health and wellbeing (either positively, or negatively)? Select all that apply

☐ Administrative staff

☐ Team-mates

☐ Opposition players

☐ Coaches

☐ Supporters

☐ Match officials

☐ Parents/Guardians

☐ Other (please specify) ________________

End of Block: Health and Wellbeing Perceptions

Start of Block: Participation

Is there anything that would make you (or would have made you) play more often?

◯ Yes

◯ No

Display This Question:

If Is there anything that would make you (or would have made you) play more often? = Yes

What factors would influence your decision to play more often? Please select all that apply

▢ More flexibility in terms of participation options

▢ Cost

▢ Less injurious

▢ Less travel

▢ Other (please specify) ____

Display This Question:

Do you currently play a form of rugby union = No

Have you previously played a form of rugby union = Yes

What were the factors that led to you stopping to play? Please select all that apply

- ☐ Time
- ☐ Age
- ☐ Cost
- ☐ Lack of flexibility in terms of participation options
- ☐ Injury concerns
- ☐ Health issues associated with rugby
- ☐ Health issues **NOT** associated with rugby
- ☐ Other (please specify) ____________

End of Block: Participation

Start of Block: Mental Health and Wellbeing

Please select the most appropriate response to the following questions

	All of the time	Most of the time	Some of the time	A little of the time	None of the time

In the past 4 weeks, about how often did you feel tired out for no good reason?	○	○	○	○	○
In the past 4 weeks, about how often did you feel nervous?	○	○	○	○	○
In the past 4 weeks, about how often did you feel so nervous that nothing could calm you down?	○	○	○	○	○
In the past 4 weeks, about how often did you feel hopeless?	○	○	○	○	○
In the past 4 weeks, about how often did you feel restless or fidgety?	○	○	○	○	○
In the past 4 weeks, about how often did you feel so restless you could not sit still?	○	○	○	○	○
In the past 4 weeks, about how often did you feel depressed?	○	○	○	○	○

In the past 4 weeks, about how often did you feel that everything was an effort?	◯	◯	◯	◯	◯
In the past 4 weeks, about how often did you feel so sad that nothing could cheer you up?	◯	◯	◯	◯	◯
In the past 4 weeks, about how often did you feel worthless?	◯	◯	◯	◯	◯

Please select the response that best describes your experiences of each over the last two weeks

	None of the time	Rarely	Some of the time	Often	All of the time
I've been feeling optimistic about the future	◯	◯	◯	◯	◯

I've been feeling useful	○	○	○	○	○
I've been feeling relaxed	○	○	○	○	○
I've been dealing with problems well	○	○	○	○	○
I've been thinking clearly	○	○	○	○	○
I've been feeling close to other people	○	○	○	○	○
I've been able to make up my own mind about things	○	○	○	○	○

Please answer the following questions:

Not at all Completely

0 1 2 3 4 5 6 7 8 9 10

Overall, how satisfied are you with life nowadays?	
Overall, to what extent do you feel that the things you do in your life are worthwhile?	
Overall, how happy do you feel?	
Overall, how anxious do you usually feel?	

Please respond to the following statements

Strongly Disagree Strongly Agree

0 1 2 3 4 5 6 7 8 9 10

I have a sense of direction and purpose in life	
Overall, I expect more good things to happen to me than bad	
If something can go wrong for me, it will	

Are you concerned about the long-term impact of rugby participation on your physical health?

◯ Yes

◯ No

Are you concerned about the long-term impact of rugby participation on your mental health and wellbeing?

◯ Yes

◯ No

From which source(s) do you/would you derive information regarding the potential impact of rugby participation on your health and wellbeing? Please select all that apply

◯ Scientific papers

◯ Materials from World Rugby or National Governing Body (e.g. RFU/IRFU)

◯ Social media

◯ Online educational modules

◯ Media

◯ Coaching staff or clubs

◯ Other (please specify) __________

From which source(s) would you ideally derive information regarding the potential impact of rugby participation on your health and wellbeing? Please select all that apply

◯ Scientific papers

◯ Materials from World Rugby or National Governing Body (e.g. RFU/IRFU)

◯ Social media

◯ Online educational modules

◯ Media

◯ Other (please specify) __________

End of Block: Mental Health and Wellbeing

Thanks for completing the survey. If you would like to receive a copy of the report from this survey, please email the lead researcher (s1135547@ed.ac.uk).

End of Block: Close
